# Incidental Pulmonary Actinomycosis in a Patient With a Pancreatic Pseudocyst

**DOI:** 10.7759/cureus.83696

**Published:** 2025-05-07

**Authors:** Nikki Anne M Ballelos, Muhammad Awan, Thomas Dao, Alison Shames, Maged Ghaly

**Affiliations:** 1 Research, Alabama College of Osteopathic Medicine, Dothan, USA; 2 Medicine, Alabama College of Osteopathic Medicine, Dothan, USA; 3 Psychiatry, Alabama College of Osteopathic Medicine, Dothan, USA; 4 Internal Medicine, Premier Medical Associates, The Villages, USA

**Keywords:** actinomycosis, bronchoscopy, pancreatic pseudocyst, pleural effusion, pulmonary actinomycosis

## Abstract

Pulmonary actinomycosis is a rare manifestation of *Actinomyces* infection and is linked to aspiration, poor oral hygiene, and respiratory disease. Although timely treatment leads to favorable outcomes, pulmonary *Actinomyces* infection often presents with nonspecific symptoms and imaging findings, delaying appropriate interventions. Our 57-year-old female patient with a history of oral cancer and chronic pancreatitis presented to the emergency department with anorexia, weakness, nausea, and diarrhea. Chest and abdominal imaging raised concerns for pancreatic disease and, to our interest, an upper lobe pleural effusion. The patient was diagnosed with a pancreatic pseudocyst, and further pulmonological testing led to the additional diagnosis of pulmonary actinomycosis, ultimately confirmed by periodic acid-Schiff staining. The pulmonary infection was an incidental finding that proved to be crucial for the patient’s recovery. This case report underscores the need for further research into pulmonary actinomycosis to identify potential patterns in its clinical presentation and imaging, with the aim of improving diagnostic techniques and time to appropriate interventions.

## Introduction

Actinomycosis is a rare bacterial infection caused by the *Actinomyces* species, which are Gram-positive, rod-shaped bacteria that may be mistaken for fungi. *Actinomyces* spp. are normal flora of the human oral cavity, colon, and genital tracts that can cause opportunistic infections in immunocompromised individuals or when integumentary barriers are breached. The four primary subtypes of actinomycosis include cervical-oral (60%), abdominal (20%), pulmonary (15%), and pelvic (5%) [1]. Diagnosis of actinomycosis is made via bacterial culture and Gram stain, revealing yellow sulfur granules and filamentous Gram-positive fungal-like pathogens consistent with *Actinomyces* [2]. Typically, infections caused by *Actinomyces* present in the cervicofacial form with soft tissue swelling of the peri-mandibular area, usually described as “lumpy jaw”, after oral surgery or in patients with poor dental hygiene [3]. Pulmonary actinomycosis is a rarer presentation of the infection and is theorized to be due to periodontal disease or aspiration of oropharyngeal secretions containing *Actinomyces*. Symptoms of the pulmonary form are nonspecific and have symptoms similar to other chronic pulmonary diseases and malignancies such as cough, sputum, and chest pain [4]. In this case report, we discuss a patient who presented with an incidental pulmonary *Actinomyces* infection while being evaluated for nonspecific symptoms initially suggestive of gastrointestinal origin.

## Case presentation

**Figure 1:**
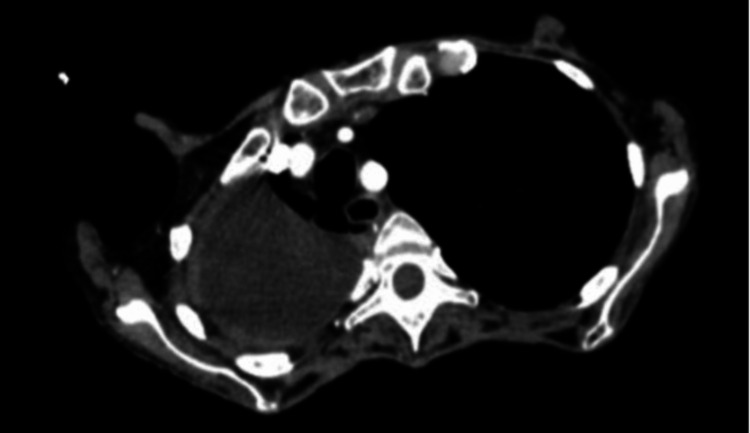
CT chest imaging demonstrating a loculated right upper lobe pleural effusion, necessitating further pulmonological testing

A 57-year-old woman presented to the emergency department (ED) with generalized weakness, dyspnea, worsening anorexia, nausea, diarrhea, and significant weight loss of about 21 pounds within two months. She had a medical history significant for hypertension, supraventricular tachycardia, gastrointestinal reflux disease, daily alcohol use, 30-pack-year smoking history, chronic pancreatitis, and retro-molar squamous cell carcinoma post-resection with a history of radiation therapy. On the physical exam, she was cachectic and appeared ill. A chest X-ray performed in the hospital was notable for volume loss of the right lung and a moderate-sized, loculated right pleural effusion. The patient was started on intravenous (IV) azithromycin and ceftriaxone (Rocephin) to provide broad-spectrum coverage of a potential underlying infection. On the same day of admission, computed tomography (CT) imaging of the patient’s chest, abdomen, and pelvis displayed a large right upper lobe pleural effusion with pleural thickening (Figure 1).

**Figure 2:**
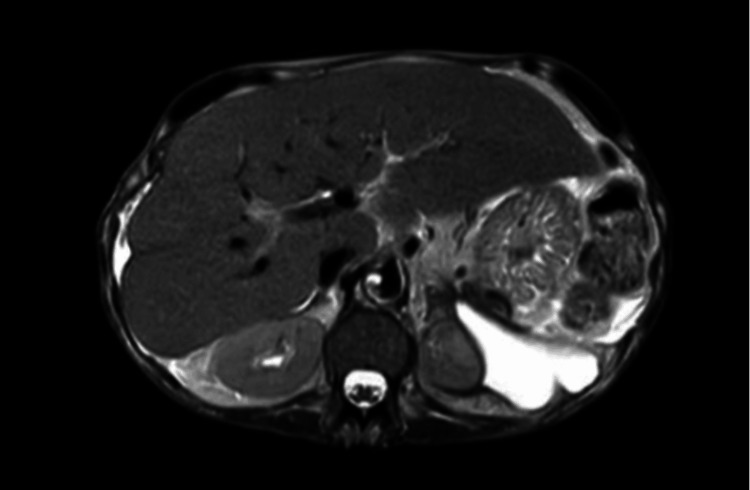
T2-weighted MRI showing a chronic pseudocyst in the left retroperitoneal space, causing mass effect upon the superior pole of the left kidney

Pancreatic calcifications and a left retroperitoneal mass were also visualized on CT of the abdomen, suggesting chronic pancreatitis with a pancreatic abscess. The retroperitoneal mass was determined to be chronic pseudocyst formation based on magnetic resonance imaging (MRI) findings on Day 2 (Figure 2).

On Day 3, pulmonology performed a diagnostic thoracentesis of the loculated pleural effusion for examination of the fluid etiology and cytology. The fluid was determined to be exudative. The smear predominantly showed lymphocytic population, mostly arranged loosely with rare clumps. Some cells appeared degenerated and few cells showed clumped chromatin and were enlarged. There was no evidence of mesothelial cells or epithelial malignancy. Due to the evidence of lymphocytic populations with fewer mesothelial cells, tuberculosis was included in the differential diagnosis. The patient was scheduled for bronchoscopy as the pleural fluid cytology was negative.

Six days later (Day 9), bronchoscopy was performed, wherein samples from the bronchoscopy were collected and sent for culture and pathology. Fiberoptic bronchoscopy visualized mucoid secretions from the right lower lobe. Bronchoalveolar lavage and biopsy of the right lower lobe were performed and sent for cultures, including acid-fast bacillus (AFB) testing and cytology. Another specimen was sent for tissue culture. The lavage culture results displayed moderate growth of normal respiratory flora, with the Gram stain showing moderate Gram-positive rods. AFB testing was negative for acid-fast bacilli. The biopsy pathology report, received on Day 11 of admission, was notable for consolidated lung parenchyma with foci of granulomatous inflammation. Furthermore, periodic acid-Schiff (PAS) fungus stain demonstrated “sulfur granules”, highly suggestive of actinomycosis.

After confirmation of the pulmonary *Actinomyces* infection, IV ceftriaxone 2 grams every 24 hours was initiated on Day 15 of admission; six weeks of IV ceftriaxone would be followed by six months of oral amoxicillin-clavulanate. After maintaining stable vitals during the rest of her hospital stay, the patient was discharged to a skilled nursing facility where she was receiving IV treatment for her actinomycosis. The patient was also scheduled to follow up with gastroenterology, pulmonology, and infectious disease.

## Discussion

We presented a 57-year-old female patient who was initially admitted for evaluation of nonspecific symptoms and significant weight loss. Imaging and further testing were consistent with a chronic pancreatic pseudocyst, a possible result of chronic pancreatitis. However, CT imaging also demonstrated suspicious pulmonary manifestations, necessitating further evaluation of the lung pathology. Samples collected from bronchoscopy were sent in for culture and staining, and the pathology report findings were highly indicative of incidental pulmonary actinomycosis, the true focus of our case study. The pulmonary manifestation of *Actinomyces* infection is rare and only constitutes 15% of all actinomycosis infections. Pulmonary actinomycosis occurs in all ages, presenting on average around 50 years of age [5,6]. *Actinomyces* involves multiple abscesses that form sinus tracts, which drain pus containing yellow granules, otherwise known as sulfur granules.

Respiratory disease (e.g., chronic bronchitis, bronchiolitis), periodontal disease, and poor oral hygiene are widely recognized risk factors for pulmonary actinomycosis. For example, a case study by Farrokh et al. presented a 66-year-old male patient with poor oral hygiene and dental disease, which may have caused his pulmonary actinomycosis [5]. Our patient had a history significant for tobacco use and retro-molar squamous cell carcinoma, suggesting her history of oral cancer and poor oral care as a possible etiology of her *Actinomyces* infection. Aspiration has also been theorized to serve a significant role in the pathogenesis of the disease. Aspiration is a complication of alcohol intoxication due to the impairment of cough and gag reflexes; in our 57-year-old patient with a known history of daily alcohol use, aspiration may be another potential cause of her pulmonary actinomycosis.

Our patient presented with generalized weakness, weight loss, and shortness of breath, which are nonspecific findings that make it difficult to confidently determine whether her symptoms were related to the *Actinomyces *infection, the pancreatic pseudocyst, or both. Potential symptoms of actinomycosis seen in the literature include anorexia [7], productive cough with white phlegm [8], fever [8], and respiratory acidosis necessitating mechanical intubation [6]. However, pulmonary *Actinomyces* infection does not produce unique physical exam findings, and not all symptoms are consistently seen. For example, weight loss may be the only manifestation of the disease [7].

Imaging for pulmonary actinomycosis is also nonspecific and therefore not diagnostic, as the infection may present with a wide range of radiographic findings on X-ray, CT, and MRI [5]. For example, CT imaging of our patient demonstrated a large right upper lobe pleural effusion with pleural thickening. This differs from findings in a study of a 64-year-old male patient by Khatib et al., who developed organizing pneumonia as a result of pulmonary *Actinomyces* infection. Pulmonary actinomycosis in their patient manifested as symmetric bilateral ground-glass opacities with interstitial thickening [6]. Another example is the 68-year-old patient in a case report by Ferreira et al., in whom pulmonary actinomycosis was shown on CT imaging as a parenchymal consolidation in the anterior segment of the right upper lobe [7]. Ding et al. discussed a 70-year-old male patient who was admitted for intermittent productive cough; pneumonia and left-sided pleural effusion were found on X-ray, with the ultimate diagnosis being pulmonary *Actinomyces* infection [8].

Due to its vague and nonspecific pulmonary symptoms and imaging findings, pulmonary actinomycosis is oftentimes misdiagnosed for other diseases (e.g., tuberculosis, lung cancer), consequently delaying necessary interventions and treatment. In the case of our patient, pulmonary *Actinomyces* infection was not confirmed until 11 days after admission and appropriate antibiotic treatment began 15 days after admission, as a result of the confusing nature of pulmonary actinomycosis. Bronchoscopy and video-assisted thoracoscopy are useful in the diagnosis of pulmonary actinomycosis. However, bronchoscopy of actinomycosis may still demonstrate variable results, including occlusion of the upper lobe bronchus by an irregular, granular thickening [5], stenosis of the anterior segment of the upper lobe bronchus [7], necrotic neoplasm obstructing the left lower lobe [8], or, as seen in our case, mucoid secretions from the right lower lobe. Therefore, biopsies of discovered lesions are diagnostic; sulfur granules on PAS stain are highly characteristic of actinomycosis and a hallmark of its diagnosis.

Penicillin is the drug of choice in the treatment of pulmonary actinomycosis, as *Actinomyces* is very susceptible to beta-lactams [6,7]. Unnecessary surgery is prevented with thorough treatment, and the prognosis is favorable in patients treated in a timely manner. Nonetheless, the response to antibiotic treatment is slow and therefore must be monitored with serial CT or plain radiographs [5]. The literature recommends a regimen of 2-6 weeks of IV beta-lactam (e.g., penicillin, ceftriaxone) followed by six months of oral amoxicillin-clavulanate [6,7], to which our 57-year-old patient demonstrated an excellent response.

## Conclusions

This case report ultimately highlights the urgency for further research into pulmonary actinomycosis to discover potential patterns in clinical presentation and diagnostic imaging. As demonstrated in our case, the nonspecific nature of the disease can hinder early diagnosis and appropriate intervention, as pulmonary *Actinomyces* infection often mimics other respiratory conditions such as malignancy or tuberculosis. Furthermore, this case study underscores the importance of maintaining a broad differential diagnosis, especially in patients with risk factors for actinomycosis. With increased awareness and education, the clinical course and outcomes for patients with pulmonary actinomycosis can be significantly improved.
